# The Chamber Gap Assay Is a Simple and Sensitive In Vitro Method for Studying Pancreatic Cancer-Induced Macrophage Recruitment and Morphological Alteration

**DOI:** 10.3390/biology15030240

**Published:** 2026-01-28

**Authors:** Maik Lenz, Stefanie Muliawan, Florian Nowak, Lea Miebach, Stephan Kersting, Tobias Schulze, Sander Bekeschus, Theresa Kordaß, Aydar Khabipov

**Affiliations:** 1 Department of General, Visceral, Vascular and Thoracic Surgery, University Medicine Greifswald, 17475 Greifswald, Germany; 2 Leibniz Institute for Plasma Science and Technology (INP), 17489 Greifswald, Germany; 3 Department of Dermatology, University Medicine Rostock, 18057 Rostock, Germany; 4 Institute of Pathology, University Medical Center and Medical Faculty Mannheim, Heidelberg University, 68167 Mannheim, Germany

**Keywords:** macrophage migration, cancer cell migration, macrophage morphology, tumor associated macrophages, pancreatic cancer, migration assay, chamber gap assay, tumor microenvironment, cancer immunology, RAW264.7, THP-1

## Abstract

Pancreatic cancer is characterized by an immunosuppressive tumor environment in which macrophages are recruited and reprogrammed to support tumor growth. Studying macrophage migration and polarization is crucial for understanding disease progression and identifying therapeutic targets. However, existing in vitro methods such as the transwell assay provide limited spatial resolution and do not allow visualization of cell movement or morphological changes. Here, we established and evaluated the Chamber Gap Assay, a modified two-compartment culture system that enables direct, time-resolved observation and quantification of macrophage migration toward pancreatic cancer cells as well as phenotypic alterations. Using murine and human macrophage–cancer cell models, we compared the performance of the Chamber Gap Assay with the transwell assay. We found that macrophage monocultures displayed substantial spontaneous migration in the transwell system, while cancer cells induced only modest additional macrophage recruitment. In contrast, the Chamber Gap Assay demonstrated clear and highly significant directional macrophage movement toward cancer cells, with distinct migration patterns and improved sensitivity for detecting group differences. The method also enabled visualization of cancer cell movement within the same system. Furthermore, CGA offers observations of morphological changes in immune cells under the influence of pancreatic cancer cells. Our findings indicate that the Chamber Gap Assay provides a robust and physiologically relevant method for studying tumor-induced immune cell recruitment and associated morphological changes.

## Simple Summary

Pancreatic cancer ranks among the most lethal malignancies. A key contributor to its poor prognosis is the capacity of tumor cells to recruit macrophages and reprogram them into a tumor-promoting rather than tumor-eliminating phenotype. Therefore, elucidating how and why macrophages are recruited to the tumor is essential for designing new therapeutic strategies. However, widely used laboratory methods do not permit direct visualization of cell movement or accurate quantification of migration dynamics. Here, we adapted and evaluated a novel in vitro method, the Chamber Gap Assay (CGA). By culturing cancer cells and macrophages in separate but connected compartments, this system permits real-time visualization of macrophage migration toward cancer cells and the resulting morphological changes. This approach enables more accurate quantification of migration compared with conventional methods. Using this approach, we showed that pancreatic cancer cells strongly attract macrophages and demonstrated that the CGA method is highly sensitive in detecting tumor-induced immune recruitment and morphological alteration in macrophages. The assay offers an accessible platform to study how tumors manipulate immune cells and may facilitate the development of therapies that target pathological immune cell recruitment.

## 1. Introduction

Pancreatic ductal adenocarcinoma (PDAC) is one of the most aggressive malignancies, with a five-year survival rate below 10% and an increasing global incidence [1,2]. Its poor prognosis is largely driven by late-stage diagnosis, early metastatic spread, and resistance to both chemotherapy and immunotherapy [3]. The tumor microenvironment (TME) in PDAC is highly immunosuppressive, characterized by dense stromal fibrosis, fibroblast activation, and significant infiltration by immune cells [4,5]. Among the infiltrating immune cells, tumor-associated macrophages (TAMs) are particularly abundant, comprising up to 50% of the tumor mass [6]. TAMs are derived from circulating monocytes recruited into the TME through chemokine gradients, notably involving signaling axes such as CCL2, CCL5, CCL17, CCL22, and CSF-1 [7,8,9,10]. Upon recruitment, TAMs adopt an M2-like phenotype, characterized by the secretion of anti-inflammatory cytokines such as IL-10, TGF-β, and VEGF, which support angiogenesis, extracellular matrix remodeling, and immune evasion [11,12,13]. The presence of TAMs in PDAC is associated with poor clinical outcomes and resistance to therapy [14]. Consequently, strategies aimed at blocking TAM recruitment or reprogramming their phenotype have emerged as a major focus in preclinical cancer research [15,16].

Macrophage infiltration into the TME plays a pivotal role in tumor progression. Two essential processes underlying immune cell recruitment are chemotaxis and chemokinesis. Chemotaxis refers to the directed movement of cells along a chemotactic gradient, whereas chemokinesis describes non-directional, random cell movement [17]. Distinguishing between these forms of migration is fundamental for understanding how immune cells respond to tumor-derived cues. In PDAC, macrophages predominantly migrate toward tumors in a chemotactic manner, driven by soluble factors secreted within the TME [18]. However, macrophages also exhibit a degree of spontaneous, non-directional motility [19]. In vitro assays are essential tools for studying the mechanisms that govern TAM recruitment and for screening potential therapeutic targets. The traditional transwell (Boyden chamber) assay, developed in the 1960s, remains widely used. However, this method lacks real-time imaging and spatial resolution, limiting its ability to reveal detailed migration patterns and cell–cell interactions [20]. Alternative systems—including scratch assays, Dunn chambers, and microfluidic devices—offer varying levels of control over chemokine gradients, throughput, and technical complexity [21,22,23,24]. Each method has distinct advantages and limitations, particularly when it comes to capturing the dynamic nature of cell migration.

## 2. Materials and Methods

### 2.1. Cell Culture

Murine PDAC cell lines PDA6606 (kind gift from Prof. David Tuveson, Cold Spring Harbor Laboratory, Cold Spring Harbor, NY, USA) and Panc02 (CLS Cell Lines Service, Heidelberg, Germany) were cultured alongside RAW264.7 macrophages (ATCC TIB-71). Human PDAC cell lines PA-TU-8988T and PA-TU-8988S (DSMZ, Braunschweig, Germany) were co-cultured with THP-1 cells (Sigma-Aldrich, St. Louis, MO, USA) as a macrophage model.

All cell lines were maintained in Dulbecco’s modified Eagle medium (DMEM; Gibco, Waltham, MA, USA) supplemented with 10% fetal calf serum (FCS) (Sigma-Aldrich, St. Louis, MO, USA) and 1% penicillin-streptomycin (Thermo Fischer Scientific, Waltham, MA, USA). All cells were incubated at 37 °C in a humidified atmosphere containing 5% carbon dioxide. Tumor cells were cultured in adherent flasks, while RAW264.7 cells were maintained in suspension flasks.

### 2.2. Transwell Migration Assay

Transwell assays were performed using ThinCert™ inserts (24 wells, 3 µm pore size; Greiner Bio-One, Frickenhausen, Germany) placed in CELLSTAR^®^ 24-well plates (Greiner Bio-One, Frickenhausen, Germany) pairing murine RAW264.7 macrophages with murine PDA6606 or Panc02 cancer cells.

Before seeding RAW264.7 macrophages in the upper compartment, cells were stained with CellTrace™ Far Red (Thermo Fisher Scientific, Waltham, MA, USA) according to the manufacturer’s protocol. To assess macrophage migration in the absence of tumor-derived chemotactic signals, a dilution series of RAW264.7 macrophages was prepared. Starting with 8 × 10^5^ macrophages, decreasing densities (4 × 10^5^, 2 × 10^5^, 1 × 10^5^, 5 × 10^4^, and 2.5 × 10^4^) of cells were seeded in the lower chambers of the transwell system to determine the maximum fluorescence intensity corresponding to these cell numbers. An independent experiment was conducted, where the same dilution series of macrophages was applied to the upper chambers without placing cancer cells in the lower compartment. Both setups were tested under two nutritional conditions: with fetal calf serum (FCS) in the medium and without FCS. Fluorescence was quantified after 24 h, enabling the assessment of baseline macrophage migration. To examine pancreatic cancer-induced macrophage migration, 2 × 10^5^ labeled RAW264.7 macrophages in 500 µL of serum-free DMEM were seeded in the upper compartment. In the lower compartment, 2 × 10^5^ Panc02 or PDA6606 cells were seeded in 500 µL of serum-free DMEM. Plates were incubated for 24 h. After removal of the upper chambers, wells were centrifuged (100× *g*, 3 min) to pellet non-adherent macrophages before fixation with 4% paraformaldehyde. Fluorescence intensity was measured using an Infinite M200 Pro plate reader (Tecan, Männedorf, Switzerland) at excitation/emission wavelengths of 630/660 nm. Each condition was performed in triplicate.

### 2.3. Establishment of the Chamber Gap Assay (CGA)

The CGA represents a modified protocol based on the ibidi© Culture-Insert 2 well system (ibidi GmbH, Gräfelfing, Germany), specifically adapted for studying directional macrophage migration toward pancreatic cancer cells. This method enables live cell tracking of immune cell migration over time in a defined and reproducible setup, suitable for both qualitative and quantitative assessments.

Murine RAW264.7 macrophages and the murine pancreatic cancer cell lines PDA6606 and Panc02 were used to establish the CGA. The model was validated for murine peritoneal macrophages of C57BL/6J mice and PDA6606 and Panc02 cells as well as human THP-1 cells and human pancreatic cancer cell lines PA-TU-8988T and PA-TU-8988S.

#### 2.3.1. Isolation of Peritoneal Macrophages byCD11b Magnetic-Activated Cell Sorting

Peritoneal cells were collected from adult C57BL/6J mice (*n* = 3) by lavage with ice-cold PBS for magnetic-activated cell sorting (MACS). Cells were pelleted (300× *g*, 5 min, 4 °C) and resuspended in MACS buffer (PBS, 0.5% BSA, 2 mM EDTA). CD11b^+^ peritoneal macrophages (PMs) were enriched using CD11b microbeads (Miltenyi Biotec, Bergisch Gladbach, Germany) according to the manufacturer’s instructions.

#### 2.3.2. Flow Cytometry

To assess the purity of MACS-isolated peritoneal macrophages, flow cytometry was performed on unfractionated lavage cells (pre-MACS), CD11b^+^ fractions, and CD11b^−^ flow-through. Cells were stained with anti-CD11b (clone M1/70, BioLegend, San Diego, CA, USA) and analyzed after the exclusion of doublets and cell debris. Purity was expressed as the percentage of CD11b^+^ cells among viable singlets.

#### 2.3.3. Isolation of Bone Marrow-Derived Macrophages

Bone marrow cells were isolated from adult C57BL/6J mice. Femurs and tibias were flushed with sterile PBS using a syringe and needle, and the cell suspension was passed through a 70 µm cell strainer. Cells were pelleted (300× *g*, 5 min) and incubated in DMEM/F-12 (Thermo Fischer Scientific, Waltham, MA, USA) supplemented with 10% fetal calf serum (FCS) and 1% penicillin/streptomycin. After 5 days of culture, non-adherent cells were removed by discarding the supernatant, and adherent macrophages were washed with PBS and collected. These bone marrow-derived macrophages were subsequently used for the chamber gap assay.

#### 2.3.4. Preparing THP-1 Monocytes for CGA

THP-1 monocytes were differentiated into more adherent (M0) macrophage-like cells by stimulation with phorbol-12-myristate-13-acetate (PMA) for 72 h (2 × 10^6^ THP-1 cells in 20 mL of RPMI with 1 ng/mL PMA; Sigma-Aldrich, St. Louis, MO, USA).

Cells were collected, washed with RPMI, pelleted (280× *g*, 5 min), and re-seeded in PMA-free RPMI for 24 h before use in the CGA. A cell suspension containing 10 × 10^6^ PMA-stimulated THP-1 cells was pelleted (280× *g*, 5 min), supernatants were removed, and cells were resuspended in 10 mL of DPBS with 10 µL of CellTrace Far Red dye (Thermo Fisher Scientific, Dreieich, Germany). The suspension was incubated for 20 min at 37 °C and 5% carbon dioxide. After staining, cells were pelleted again, washed, resuspended in 10 mL of RPMI, and maintained at 4 °C until use.

#### 2.3.5. Experimental Setup of CGA

A sterile ibidi Culture-Insert 2 well was placed in the center of a µ-dish, 35 mm, high (ibidi GmbH, Gräfelfing, Germany), creating two separate compartments. In one chamber, 2 × 10^4^ tumor cells (murine Panc02 and PDA6606 cells, as well as human PA-TU-89988T and PA-TU-8988S cells) were seeded; in the opposite chamber, macrophages were seeded in 70 µL of complete DMEM with 10% FCS (5 × 10^4^ RAW264.7 macrophages, 5 × 10^4^ murine bone marrow macrophages, 3 × 10^4^ murine peritoneal macrophages, or 5 × 10^4^ THP-1). Negative controls consisted of macrophages seeded opposite an empty compartment. THP-1 cells were already stained during PMA differentiation; murine macrophages were stained separately with CellTrace™ Far Red (Thermo Fisher Scientific, Waltham, MA, USA), while pancreatic cancer cells were stained with Tag-it Violet™ (BioLegend, San Diego, CA, USA), enabling dual-color fluorescence discrimination. All cells were stained for 30 min at 37 °C in 750 µL of dye-containing PBS (20 µM Tag-it Violet, 4 µM CellTrace Far Red) and washed in pre-warmed medium afterwards.

**Figure 1:**
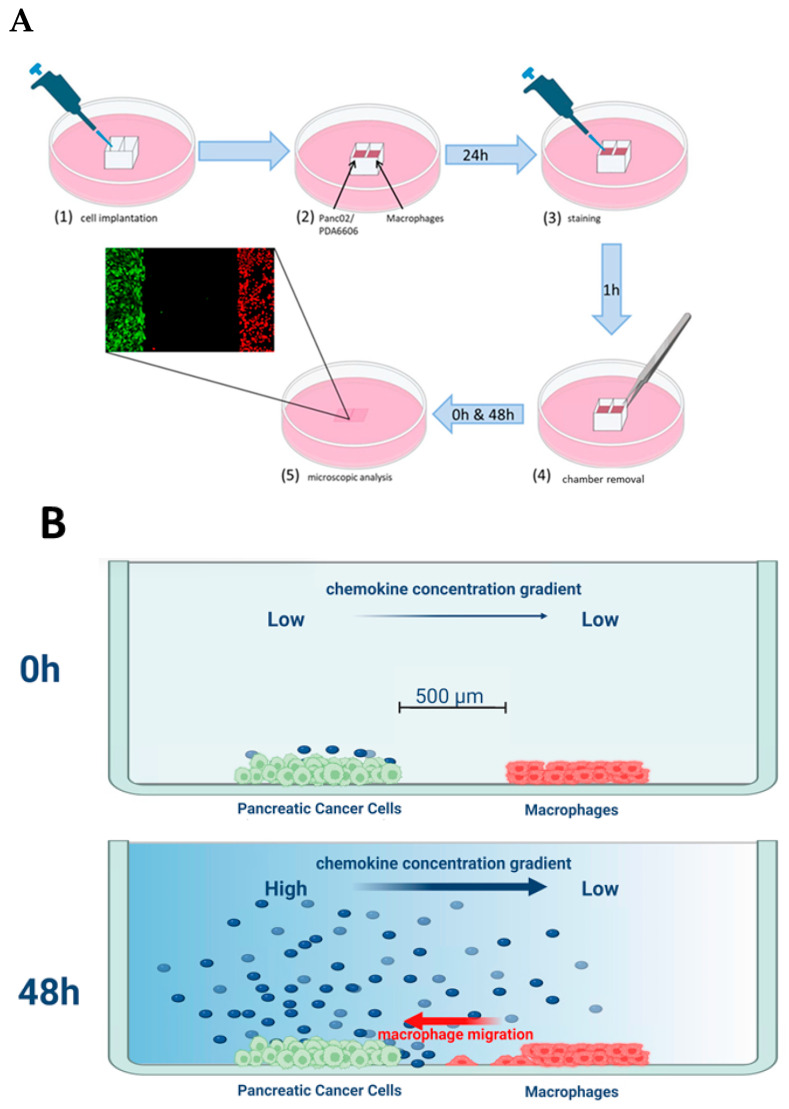
Schematic overview of the Chamber Gap Assay (CGA). (**A**) A sterile ibidi Culture-Insert 2 well was placed in the center of a µ-dish 35 mm, high, generating two adjacent compartments. One chamber was seeded with 20,000 murine PDAC cells (Panc02 or PDA6606 cell lines), and the opposite chamber with 5 × 10^4^ RAW264.7 macrophages in 70 µL of complete DMEM; negative controls received macrophages opposite an empty chamber. After 12 h of incubation (37 °C, 5% CO_2_), cells were fluorescently labelled (RAW264.7 macrophages: CellTrace™ Far Red; tumor cells: Tag-it Violet™) and washed and 900 µL of fresh medium was added around the insert. Gentle removal of the insert yielded a reproducible cell-free gap of around 500 µm. Dishes were topped up to 2 mL of medium and briefly rinsed to eliminate detached cells. Fluorescence microscopy (BZ-9000; Keyence, Osaka, Japan) captured Brightfield, TxRed (macrophages), and DAPI (tumor cells) channels immediately after insert removal (0 h) and at 24 h, 48 h, and 120 h. (**B**) After removal of the culture insert (T_0_), the cell-free gap separated the RAW264.7/THP-1 macrophages from the tumor cells (murine Panc02/PDA6606 cells or human PA-TU-8988S/PA-TU-8988T cells). At this early time point, the chemokine concentration gradient was low as limited diffusion has occurred. Over time, tumor cells secreted increasing amounts of chemotactic factors, leading to directed migration of macrophages toward the tumor compartment.

After staining, 900 µL of fresh DMEM was added around the insert, and the insert was gently removed, generating a reproducible gap between compartments of around 500 µm width. A final wash removed floating cells, and 2 mL of fresh medium was added. The experiments were performed up to 144 h. At 120 h, a propidium iodide stain (Sigma-Aldrich, St. Louis, MO, USA) (2 µm/mL) was applied to enable the identification of necrotic cells. Schematic sketches of the experimental setup and workflow are presented in Figure 1A,B.

#### 2.3.6. Imaging and Quantification

**Figure 2:**
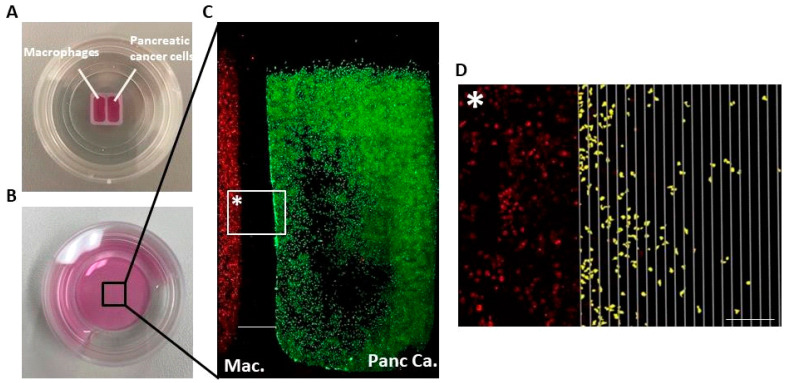
Macroscopic pictures of CGA and microscopic detection of migrated macrophages. (**A**) macroscopic image of ibidi chamber with macrophages in left chamber and tumor cells in right chamber. (**B**) Macroscopic image of well after removing ibidi chamber. (**C**) Microscopic image of field of interest after cell stain and chamber removement. Migration dynamics are visualized by fluorescence microscopy: macrophages are dyed with CellTrace™ Far Red and tumor cells with Tag-it Violet™, enabling direct observation of cell positioning and movement (scalebar = 500 µm). (**D**) Image represents marked box* in image (**C**): Macrophage migration in the gap 48 h after chamber removal. Migrated cells are detected and counted per interval in Qupath© (marked yellow), quantified by number of migrated macrophages per 100 µm interval for murine cell lines ((**D**) scale bar = 100 µm. Interval ticks = 20 µm).

Fluorescence images were acquired immediately after insert removal (T0) and subsequently after 48 h for the murine cell line RAW264.7, and after 24, 48, and 120 h for primary murine macrophages as well as for human cell lines, using a Keyence BZ-9000 microscope. Macroscopic images of the CGA setup before and after ibidi© insert removal are shown in Figure 2A,B. Overview images (40× magnification) were acquired in three channels: brightfield, TxRed, and TxGreen (Figure 2C). Exposure settings were optimized for each channel (typically 1/3.5 s for macrophages and 1/2 s for tumor cells).

To ensure valid comparisons, images were aligned such that the center of the cell-free gap corresponded to the center of the image.

Quantitative analysis of migration was performed using QuPath© (version 0.4.3). The built-in cell detection algorithm was configured to identify fluorescently labeled cells and quantify migration into the gap (Figure 2D). Migration was assessed by quantifying macrophages or cancer cells per defined gap interval adjacent to the cell culture border after removal of the ibidi© insert. Migration was quantified by counting migrated cells per 100 µm intervals. The complete QuPath© workflow (1: create project; 2: add images; 3: import files; 4: set pixel size; 5: create object annotations for intervals; 6: define annotation width and height; 7: label intervals; 8: select all annotations; 9: transform annotations; 10: align to the cell border; 11: lock annotations; 12: perform cell detection; 13: set detection parameters; 14: export data) is available online and illustrated in Figure 2D.

Directional migration of peritoneal macrophages toward cancer cells (“cis-migration”) and away from them (“trans-migration”) was compared by quantifying the total number of migrated cells on both sides of the macrophage monolayer. Morphological changes of peritoneal macrophages were evaluated by analyzing high-magnification images (400×) and determining the percentage of morphologically altered macrophages displaying more than three membrane protrusions (filopodia-/lamellipodia-like structures) per field of view (200× magnification).

At 144 h, overview images of CGA co-cultures (murine peritoneal macrophages with Panc02 or PDA6606 tumor cells) were acquired for morphological culture analysis after the cancer cells had overgrown the macrophage culture area.

### 2.4. Statistical Analysis

Data were analyzed using GraphPad Prism 9. Normal distribution was assessed using the Shapiro–Wilk test. For the transwell assay, statistical significance between experimental conditions (macrophages + tumor cells (PDA6606 or Panc02)) and the control (macrophages only) was determined using Kruskal–Wallis test followed by Dunn’s multiple comparisons test.

For the Chamber Gap Assay, migration was analyzed both over time and between experimental conditions. Macrophage migration was quantified as the number of cells per distance interval at each time point. To assess differences in macrophage migration between experimental conditions (macrophages + tumor cells vs. control (macrophages only)) across the time course, data were analyzed using a two-way ANOVA test with “condition” and “time” as factors, followed by appropriate post hoc testing.

To assess differences between conditions within individual time points (0 h, 24 h, 48 h, 120 h), spatial migration patterns (cells per distance interval) were analyzed using a two-way ANOVA test with “condition” and “distance interval” as factors. For cis- versus trans-migration analyses, observed in CGA, group comparisons were performed using an unpaired two-tailed *t*-test. *p*-values < 0.05 were considered statistically significant.

Significance levels are indicated as follows: *p* < 0.05 (*), *p* < 0.01 (**), *p* < 0.001 (***), *p* < 0.0001 (****).

## 3. Results

### 3.1. Transwell Migration Assay Demonstrates Baseline RAW264.7 Migration and Serum-Dependent Enhancement by FCS

**Figure 3:**
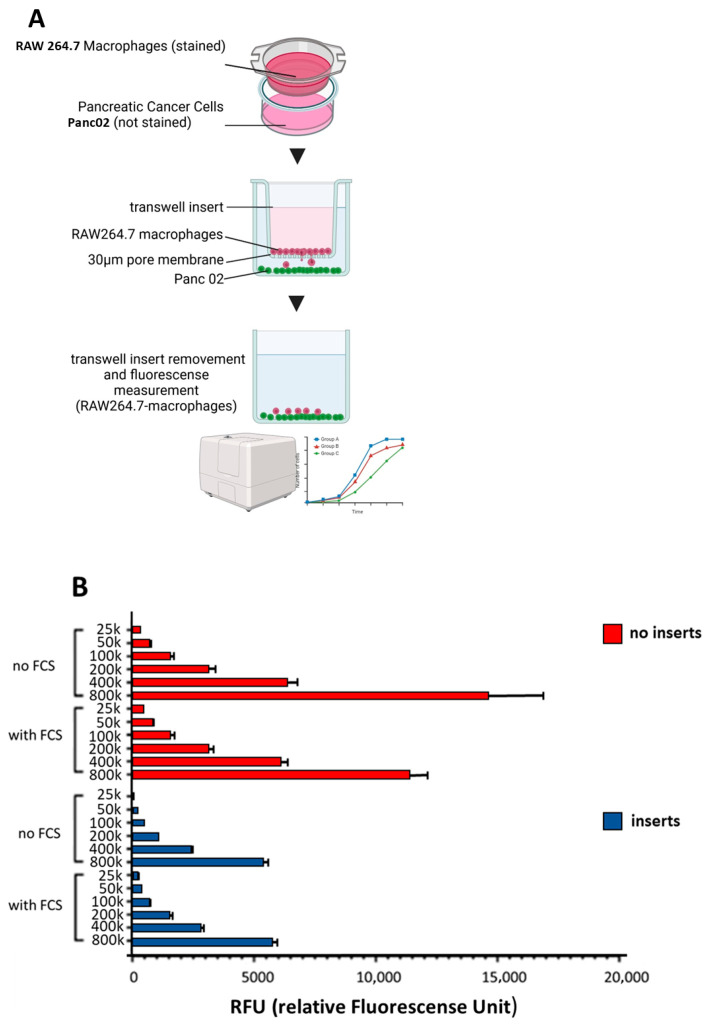

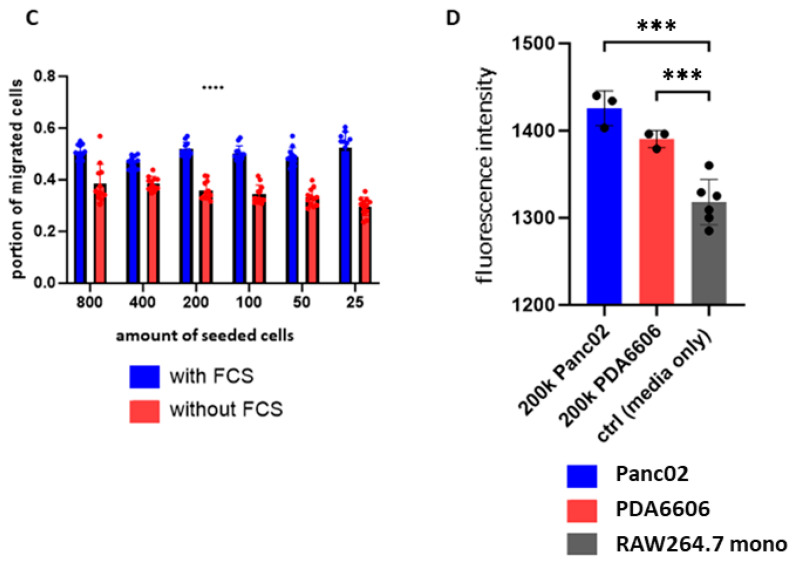
Transwell migration assay to assess tumor-induced macrophage chemotaxis. (**A**) Schematic illustration of the transwell setup. CellTrace™ Far Red-labeled RAW264.7 macrophages (2 × 10^5^) were seeded into the upper chamber of ThinCert™ inserts (3 µm pore size, 24-well format) in serum-free DMEM. The lower compartment contained either 5 × 10^5^ Panc02 cells in serum-free DMEM or serum-free DMEM alone as a negative control. (**B**) A dilution series of RAW264.7 macrophages in the lower compartment (2.5 × 10^4^ to 8 × 10^5^ cells; no insert) was used to determine the maximal fluorescence signal and to validate the proportionality between cell number and fluorescence intensity. In parallel, a dilution series of RAW264.7 macrophages was seeded in the upper compartment with no cells present in the lower compartment to assess spontaneous, cell density-dependent migration into the lower chamber. This experimental setup was used to demonstrate that native macrophage migration scales proportionally with cell number. Fluorescence of migrated macrophages was quantified after 24 h. Experiments were performed in the presence and absence of fetal calf serum (FCS) in the culture medium. (**C**) Quantification of native migrated macrophages (no cancer cells placed in lower compartment) after 24 h showing FCS as a migration trigger. **** indicated statistical difference between groups (FCS vs. no FCS). (**D**) Quantification of pancreatic cancer-induced macrophage migration with 200,000 RAW264.7 macrophages in the upper compartment and either 2 × 10^5^ Panc02 cells, 2 × 10^5^ PDA6606 cells, or no cells in the lower compartment (timepoint: 24 h). Fluorescence intensity in the lower compartment was slightly but significantly increased in wells containing Panc02 or PDA6606 cells in the lower compartment, indicating an enhanced chemotactic migration of RAW264.7 macrophages in the presence of pancreatic cancer cells. Data represent the mean ± SD of three biological replicates. Statistical significance between groups was determined using Kruskal–Wallis Test followed by Dunn’s multiple comparisons test. Significance levels are indicated as follows: *p* < 0.001 (***), *p* < 0.0001 (****).

The transwell assay (experimental setup schematically illustrated in Figure 3A) demonstrated substantial spontaneous migration of RAW264.7 macrophages in the absence of tumor cell-derived chemotactic signals. Across all tested macrophage concentrations, approximately 50% of cells migrated from the upper to the lower compartment within 24 h. When fetal calf serum (FCS) was removed from the medium to minimize potential migration stimuli, spontaneous migration was reduced to approximately 30% (Figure 3B,C). All subsequent migration assays were therefore performed under serum-free conditions.

To further restrict spontaneous macrophage movement, the transwell membrane (3-µm pore size) was coated with Matrigel at various concentrations and volumes. However, this modification did not significantly reduce the baseline migration of RAW264.7 macrophages into the lower compartment.

### 3.2. Transwell Assay Demonstrates Pancreatic Cancer Cell-Induced Enhancement of RAW264.7 Migration

In the classical transwell setup, RAW264.7 macrophages exhibited a significant increase in migration toward both Panc02 and PDA6606 pancreatic cancer cells. After 24 h, the fluorescence signal in the lower compartment reached RFU = 1426 for macrophages migrating toward Panc02 cells compared with RFU = 1318 in the empty control (*p* = 0.0003). Similarly, migration toward PDA6606 cells resulted in RFU = 1390, which was again significantly higher than the control (RFU = 1318, *p* = 0.0003). These data indicate that pancreatic cancer cells elicit a detectable chemotactic response in RAW264.7 macrophages, although the relatively small effect size highlights the pronounced intrinsic motility of this macrophage line even in the absence of tumor-derived cues (Figure 3D).

### 3.3. Chamber Gap Assay Enables Sensitive Analysis of Cancer-Induced Macrophage Migration

**Figure 4:**
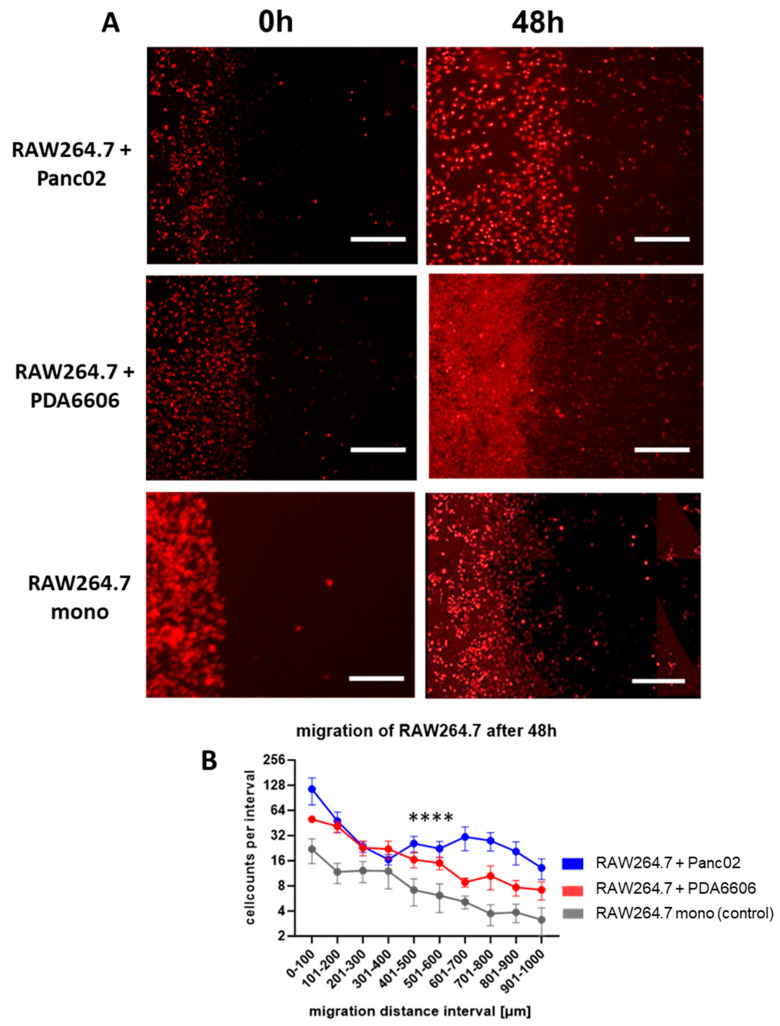
Microscopic images and quantification of migrated RAW264.7 macrophages in CGA. (**A**) Top and middle row: Empty chamber gap directly after removing chamber (left column) and after 48 h with RAW264.7 macrophages in left chamber and PDA6606 cells or Panc02 cells in right chamber (right column). Bottom row: control with RAW264.7 macrophages in left chamber and empty right chamber. After 48 h, an increase in migration could be observed in all three settings. (**B**) The quantification of macrophage migration towards PDA6606 or Panc02 cells 48 h after chamber removal shows increased migration in the presence of pancreatic cancer cells compared with the macrophage-only control setup. For statistical analysis between groups, a two-way ANOVA test was performed. (Significance level of differences between conditions: *p* < 0.0001 (****)), Scale bar = 100 µm.

The Chamber Gap Assay (CGA) enables direct visualization and quantification of macrophage migration toward pancreatic cancer cells. Following barrier removal, macrophages migrated into a ~500 µm-wide cell-free gap between the two cell compartments. Cell migration was initially demonstrated using murine RAW264.7 macrophages (Figure 4A). Comparison between control conditions (empty compartment) and the co-culture with cancer cells revealed a highly significant effect of tumor cells on RAW264.7 macrophage migration (Figure 4B). The strongest effects were observed within the first 200 µm from the original macrophage boundary, where the steepest gradients and the largest differences between experimental groups occurred (Figure 4B).

**Figure 5:**
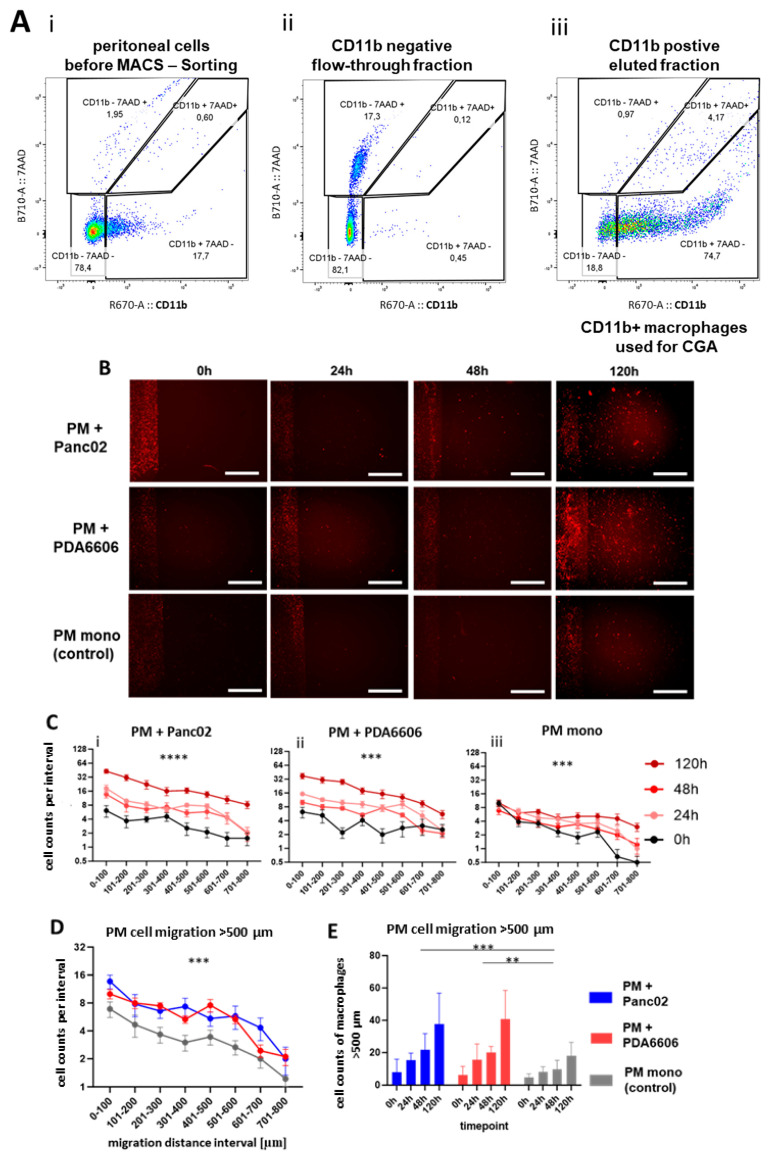
Migration of peritoneal macrophages toward pancreatic cancer cells following CD11b MACS enrichment. (**A**) Flow cytometry analysis of CD11b MACS-enriched murine peritoneal macrophages. Representative dot plots show CD11b versus 7-AAD staining of (**i**) unfractionated peritoneal lavage, (**ii**) cells CD11b^−^ flow-through, and (**iii**) CD11b^+^ magnet-eluted, confirming enrichment of viable CD11b^+^ macrophages. (**B**) Representative overview images of Chamber Gap Assays at 0, 24, 48, and 120 h. Peritoneal macrophages were seeded on one side of the chamber, while PDA6606 cells, Panc02 cells, or no cancer cells were placed on the opposite side. The fluorescence signal of cancer cells was masked to improve the visualization of macrophage distribution. Scale bars = 100 µm. (**C**) Time-dependent changes in macrophage counts per interval indicate progressive migration over 120 h under conditions (**i**) PM and Panc02, (**ii**) PM and PDA6606 and control (**iii**) PM mono. (**D**) Comparison of macrophage counts per interval at 48 h demonstrating increased migration toward PDA6606 and Panc02 cell compartments compared with empty controls. For statistical analysis, a two-way ANOVA test was performed. (**E**) Quantification of macrophages migrating ≥ 500 µm from the seeding area showing ongoing migration over time, with higher cell counts in the presence of pancreatic cancer cells. Significance levels are indicated as follows: *p* < 0.01 (**), *p* < 0.001 (***), *p* < 0.0001 (****).

To validate the assay using primary cells, murine peritoneal macrophages (PMs) were isolated from peritoneal lavage and analyzed using the CGA (Figure 5). Magnetic-activated cell sorting (MACS) increased the proportion of CD11b^+^ cells from 17.7% to 74.7% (Figure 5A). PMs migrated into the cell-free gap in a manner comparable with RAW264.7 macrophages (Figure 5B). The number of migrated PMs per distance interval increased across all measured time points (Figure 5C), indicating consistent migration dynamics both in the co-culture with Panc02 and PDA6606 cells (Figure 5(Ci,ii)) and in PM monocultures (Figure 5(Ciii)). The presence of cancer cells increased PM migration, as shown for the 48 h time point (Figure 5D). Moreover, the proportion of PM migrating distances greater than 500 µm was significantly higher in the co-culture with cancer cells compared with PM monocultures (Figure 5D).

**Figure 6:**
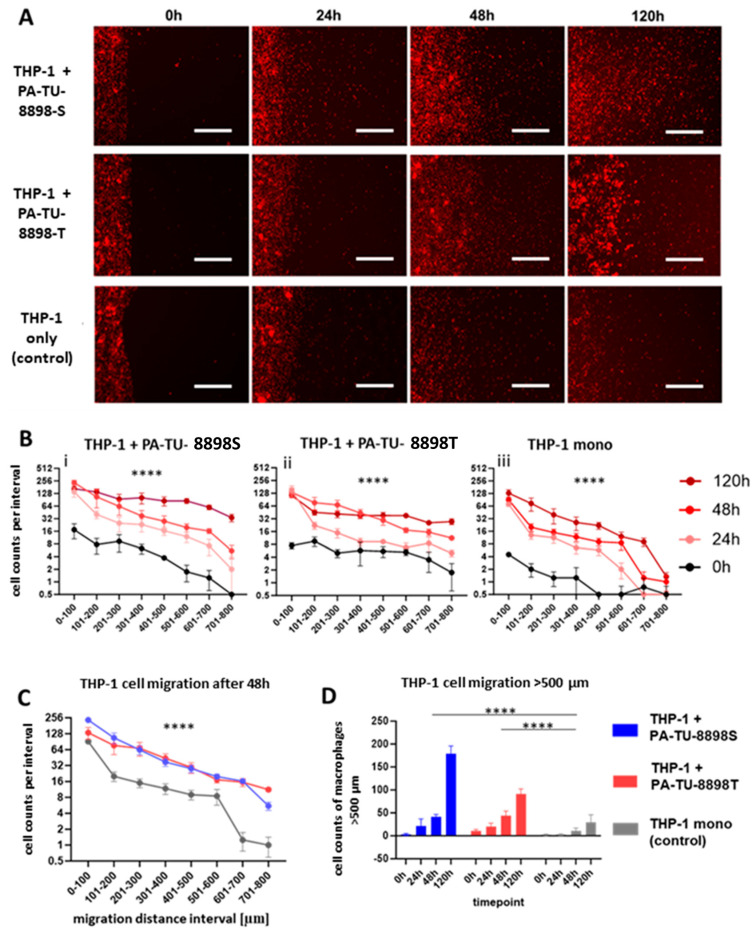
Microscopic pictures of migrated THP-1 cells and quantification of migration in CGA. (**A**) Chamber gap directly after removing chamber (0 h) and after 24, 48, and 120 h with THP-1 macrophages in the left chamber and PA-TU-8988T cells (top), PA-TU-8988S cells (middle), or no cells (bottom) in the right chamber. The fluorescence signal of cancer cells is masked. Scale bars = 100 µm. (**B**) Increasing cell counts per intervals during the observation period of 120 h indicating macrophage migration over time for conditions (**i**) THP-1 with PA-TU-8898S, (**ii**) THP-1 with PA-TU-8898 and control (**iii**) THP-1 mono. (**C**) Comparison of cell counts per interval after 48 h show a macrophage-recruiting effect of PA-TU-8988T cells and PA-TU-8988S cells, while the migration tendency towards the empty well compartment remains relatively low. Intervals for analysis were 100 µm wide. For statistical analysis, a two-way ANOVA test was performed. (**D**) Counts of THP-1 macrophages that migrated more than 500 µm, indicating ongoing migration efforts within the observational period, with an increased macrophage migration when pancreatic cancer cells are present. Data sets are attached as Table S1 (.exl). Significance levels is indicated as *p* < 0.0001 (****).

The effects of the human pancreatic cancer cell lines PA-TU-8988T and PA-TU-8988S on the migration of THP-1 macrophages (Figure 6) were comparable with those observed for murine PDA6606 and Panc02 cells on RAW264.7 macrophages and PMs. In the human cell model, we observed a time-dependent increase in THP-1 macrophage migration (Figure 6A,B), an increased migration toward pancreatic cancer cells (Figure 6C), and a gradual rise in the number of migrated THP-1 cells, including cells reaching migration distances beyond 500 µm (Figure 6D).

**Figure 7:**
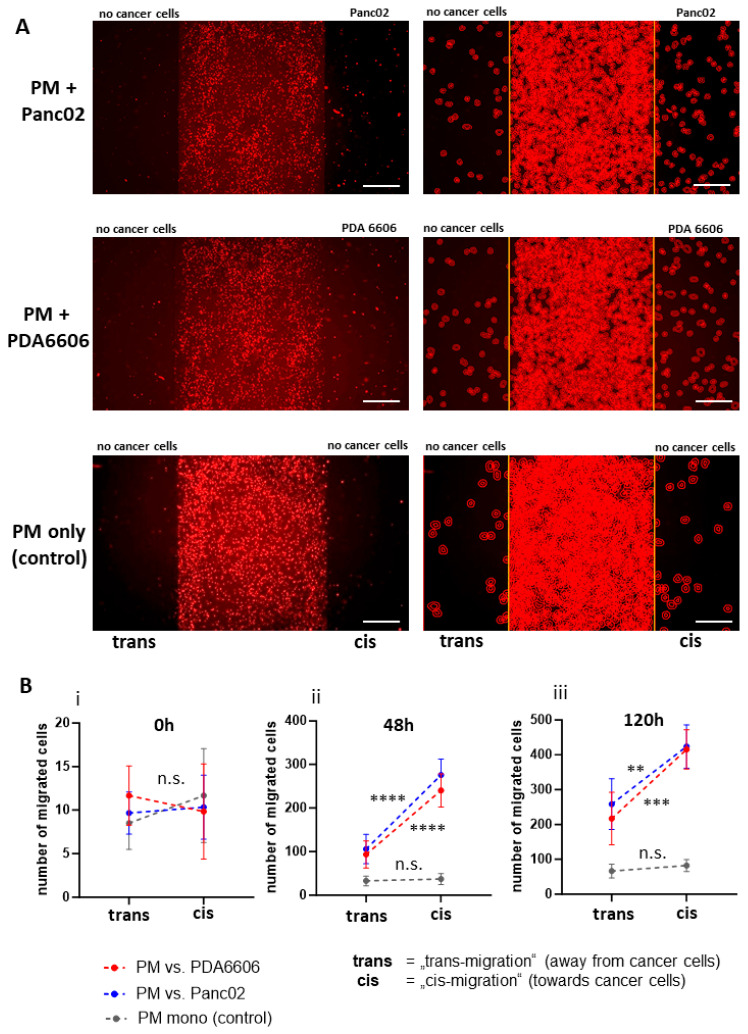
Cis- and trans-migration of macrophages in response to cancer cells. (**A**) Representative overview images illustrating macrophage distribution in the migration assay after 48 h. The left column shows the macrophage monolayer in activated fluorescence images; the right column displays the corresponding macrophage segmentation used for quantification (QuPath©). Trans-migration (migration away from the macrophage seeding area; no cancer cells present) is visualized as cell movement towards the left-hand side of the respective images, whereas cis-migration (migration toward the cancer cell compartment) is oriented towards the right-hand side of the images. Cancer cell signals were masked to facilitate the visualization of macrophage localization. Peritoneal macrophages cultured without cancer cells display low motility with an approximately symmetric distribution. In the presence of cancer cells, macrophages show a marked migration tendency towards them. Scale bars = 500 µm. (**B**) Quantification of cis- and trans-migration at indicated time points (**i**) 0 h, (**ii**) 48 h and (**iii**) 120 h after removal of ibidi© insert; Immediately after removal only a few detached cells were detected and no significant differences were observed, irrespective of the presence of cancer cells. At 48 h, a significant increase in migration toward cancer cells was detected, whereas peritoneal macrophage monocultures exhibited no directional preference and reduced overall migration. At 120 h, the same pattern was maintained, although the level of statistical significance decreased. Cis-migration vs. trans-migration was analyzed via a non-paired *t*-test. Significance levels are indicated as follows: n.s. = not significant (*p* > 0.05), *p* < 0.01 (**), *p* < 0.001 (***), *p* < 0.0001 (****).

Notably, migration was predominantly directed toward the cancer cell compartment (cis-migration), whereas migration in the opposite direction (trans-migration) was significantly reduced (Figure 7). This directional effect was evident in overview images (Figure 7A) and was confirmed by quantitative analysis using QuPath© (Figure 7B). While no directional differences were detected at time point 0 h, the most pronounced difference between cis- and trans-migration was observed after 48 h (Figure 7B). In the absence of cancer cells, no directional migration was observed and no significant differences between migration directions were detected (Figure 7B). All observed effects were statistically significant with *p*-values ≤ 0.01 (**). High-magnification images of peritoneal macrophage (PM) migrating distances greater than 500 µm showed that these cells were adherent and displayed intact morphology, suggesting preserved viability. Staining of PMs and THP-1 with propidium iodide after 120 h confirmed that macrophages remain viable during long-term co-culture (shown in Figure S1).

### 3.4. Pancreatic Cancer Cells Induce Morphological Alterations of Peritoneal Macrophages

**Figure 8:**
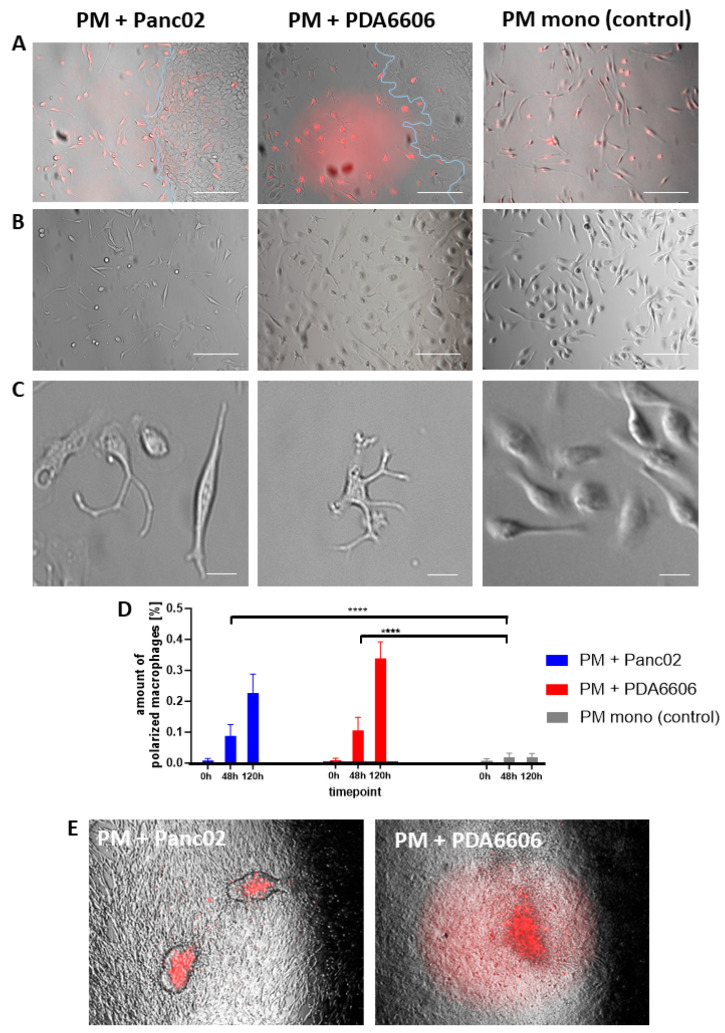
Cancer cell-induced morphological alterations of peritoneal macrophages. (**A**) Representative images of peritoneal macrophages (PMs) co-cultured in CGA with Panc02 or PDA6606 cells or PM monoculture (control) at 120 h. To discriminate macrophages from cancer cells, brightfield images were overlaid with CellTrace™ Red fluorescence from MACS-enriched PM; cancer cell borders are indicated. In the presence of cancer cells, PMs display elongated shapes and multiple membrane protrusions, whereas PM monocultures remain predominantly round. Scale bars = 50 µm. (**B**) Additional overview images at 120 h showing similar morphological alterations independent of immediate proximity to cancer cells, indicating soluble mediators as potential drivers of morphological alterations of PM. Scale bars = 50 µm. (**C**) High-magnification images illustrating characteristic morphologies. Cells with more than three cell membrane protrusions were defined as phenotypically altered macrophages. Co-cultivation with Panc02 cells induces the macrophages to elongate and develop three to four protrusions, whereas the presence of PDA6606 cells promotes the formation of macrophages with up to seven protrusions. In PM monocultures, the cells remained short and oval. Scale bars = 10 µm. (**D**) Quantification of morphologically altered PMs. The proportion of macrophages exhibiting ≥3 membrane protrusions per field of view (corresponding to panels in (**A**,**B**)) was determined. The presence of cancer cells led to a time-dependent increase in morphologically altered PMs, whereas PM monocultures showed no detectable effect. (**E**) Overview images of co-cultures at 144 h. Overgrowth of the macrophage area by cancer cells leads to formation of PM cell clusters (cell trace red positive). Cell cluster formation was not observed in naïve PM monocultures. Significance level is indicated as: *p* < 0.0001 (****).

After 120 h of CGA co-culture, peritoneal macrophages exhibited pronounced morphological alterations in the presence of tumor cells (Figure 8). The co-culture with Panc02 cells was associated with elongated cellular morphology and development of 2–4 membrane protrusions, while PDA6606 cells induced the formation of 3–7 membrane protrusions. This phenomenon was observed both in close proximity to cancer cells (Figure 8A) and within the central region of the macrophage culture (Figure 8B). High-magnification images revealed pronounced morphological heterogeneity between experimental groups. Naïve PMs predominantly exhibited a rounded to oval morphology, whereas occasional elongated cells were also observed (Figure 8C). Over time, these phenomena increased. After 140 h, approximately 25% of Panc02 cell-associated peritoneal macrophages and around 35% of PDA6606 cell-associated macrophages exhibited pronounced morphological alterations (Figure 8D). In contrast, macrophages maintained in monoculture remained predominantly rounded. As a further morphological feature of pancreatic cancer, stimulated macrophages cell cluster formation was observed when the PMs were overgrown by cancer cells (approximately after 144 h) for both the PDA6606 and Panc02 cell lines (Figure 8E). Cell cluster formation was not observed in the PM monocultures.

### 3.5. Chamber Gap Assay Detects Cancer Cell Migration

**Figure 9:**
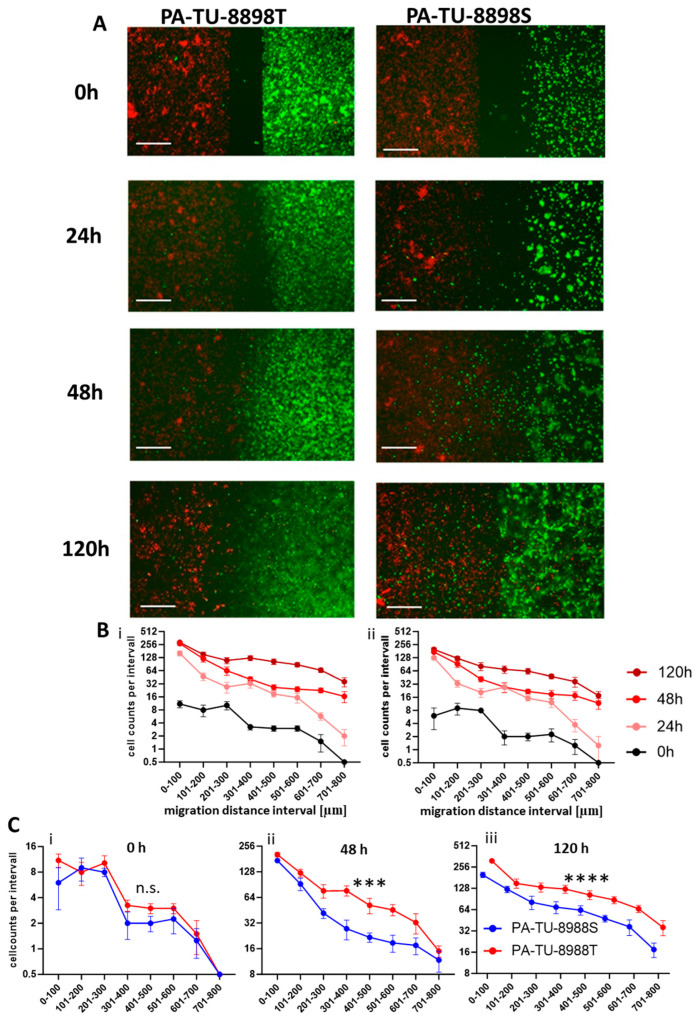
Visualization of cancer cell and macrophage migration in the Chamber Gap Assay. (**A**) Human pancreatic cancer cells (PA-TU-8988T or PA-TU-8988S) are shown in green on the right side of the gap, and PMA-differentiated THP-1 macrophages appear in red on the left side. To enhance visual clarity, the fluorescence saturation of the green channel was increased, whereas the red channel intensity was reduced. Representative images acquired at 0 h, 24 h, 48 h, and 120 h demonstrate progressive cell movement into the gap region. The images illustrate that the Chamber Gap Assay allows simultaneous visualization of tumor cell motility and immune cell recruitment within the same experimental setup. (**B**) Increasing cancer cell counts per intervals during the observation period of 120 h indicating cancer cell migration over time for both cancer cell lines (**i**) PA-TU-8898T and (**ii**) PA-TU-8898S. Both cancer cells and macrophages exhibit time-dependent migration toward the opposing compartment, with tumor cell displacement clearly observable at all time points. Macrophage migration dynamics are quantified separately in Figure 5. (**C**) Quantification of cancer cell migration at time points (**i**) 0 h (immediately after ibidi© insert removal), (**ii**) 48 h after insert removal and (**iii**) 120 h after insert removal indicating that PA-TU-8898T cells migrated more than PA-TU-8898S cells. Significance levels are indicated as follows: n.s. = not significant (*p* > 0.05), *p* < 0.001 (***), *p* < 0.0001 (****).

In addition to monitoring macrophage behavior, the Chamber Gap Assay enabled visualization of tumor cell dynamics. Both PA-TU-8988T and PA-TU-8988S pancreatic cancer cells gradually extended into the gap region over time (Figure 9A), resulting in a progressive occupation of the initially cell-free area and an increase in the number of cancer cells present in the gap at each time point (Figure 9B). PA-TU-8988T cells appeared to cover the gap more extensively than PA-TU-8988S cells, and this difference increased over time (Figure 9C).

As the experimental setup was designed to investigate cancer cell-induced macrophage recruitment, for cancer cell migration, no negative control was included to distinguish spontaneous displacement from directed migration. Therefore, the observations are reported here as descriptive evidence of cell displacement within the assay. The assay allowed reliable documentation of tumor cell dynamics under co-culture conditions.

## 4. Discussion

Tumor-associated macrophages (TAMs) are recognized as key mediators in pancreatic ductal adenocarcinoma (PDAC), contributing to tumor progression, immune evasion, and resistance to therapy. Consistent with their predominantly M2-like phenotype, TAMs exert pro-tumorigenic functions, including modulation of immune surveillance, promotion of angiogenesis, and facilitation of tumor invasion and metastasis [4,5,6,7,8,9]. Given the functional relevance of TAMs within the PDAC tumor microenvironment, there is a clear need for reliable and physiologically relevant in vitro models that allow investigation of macrophage recruitment and migration dynamics.

The results presented here suggest that the Chamber Gap Assay (CGA) represents a robust and sensitive approach for studying cancer cell-induced macrophage recruitment and associated morphological alteration. Importantly, the CGA is not intended to replace established methods but rather to serve as a useful complement to widely used assays such as the transwell (Boyden chamber) system. The transwell assay, originally described in 1962, remains a robust and accessible platform for comparative migration analyses. Nevertheless, certain technical aspects should be considered when interpreting transwell data, including baseline macrophage motility (as also observed in the present study; Figure 3C), potential gravitational effects due to the vertical setup, and the reliance on endpoint measurements, which limit the assessment of dynamic migration processes. In addition, the restricted spatial resolution hampers direct visualization of migration behavior.

By contrast, the CGA enables real-time, spatially resolved visualization of cell movement. In contrast to scratch assays, which represent another simple and cost-efficient migration model, the CGA allows two distinct cell populations to be cultured in spatially separated compartments within the same well, thereby limiting premature paracrine interactions. After removal of the barrier, a chemotactic gradient can develop under co-culture conditions, supporting macrophage migration toward tumor cells. The compartmentalized design further allows selective treatment of one cell population while maintaining the integrity of the other, which may be advantageous for mechanistic studies of macrophage recruitment [10,13]. Comparative characteristics of the Chamber Gap Assay (CGA) and the transwell assay for analyzing tumor-induced macrophage migration are shown in Table A1 (Appendix A). 

Beyond migration kinetics, the CGA also enables high-resolution analysis of macrophage morphology during migration, providing insight into different migratory phenotypes, such as amoeboid versus mesenchymal movement. Amoeboid migration is typically characterized by a rounded morphology and rapid movement in confined environments, whereas mesenchymal migration is associated with elongated morphology, increased matrix interaction, and slower but more persistent migration [25]. The visualization and quantification of cancer-induced morphological changes in peritoneal macrophages observed in this study highlight the analytical potential of the CGA and may allow future applications such as image-based machine learning approaches for macrophage subset classification [26].

In the present study, no gene or protein expression analyses (e.g., FACS or qPCR) were performed to formally distinguish M1 and M2 macrophage phenotypes. However, based on the observed morphological changes and in consideration of the existing literature, the data are consistent with a cancer cell-induced M2-like polarization of peritoneal macrophages by PDA6606 and Panc02 cells. For example, the cluster formation observed in murine peritoneal macrophages in the presence of PDA6606 or Panc02 (Figure 8E) has previously been reported in RAW264.7 macrophages and was associated with an M2-like phenotype characterized by expression of CD206 and arginase-1 [5]. In agreement with this, previous studies have shown that peritoneal macrophages from C57BL/6J mice form similar clusters when stimulated with IL-4, a key inducer of M2 polarization [27]. Moreover, PDA6606 has been shown to induce an M2-like phenotype in peritoneal macrophages of C57BL/6J mice in vivo [28]. Morphological analyses of murine peritoneal macrophage subsets further demonstrated that elongated morphology with cell protrusions is characteristic of M2 macrophages, consistent with our observations (Figure 8B,C) [29].

Collectively, these findings suggest that peritoneal macrophages acquire an M2-like phenotype in the CGA, in line with their response to tumor-derived signals reported in previous studies [30]. In this context, we have previously shown that PDA6606 and Panc02 cells induce expression of the chemokine receptors CCR2 and CCR4 in RAW264.7 macrophages [30]. CCR2 expression is associated with increased macrophage chemotaxis [18,31], while CCR4 has been linked to enhanced macrophage recruitment into the pancreatic tumor microenvironment in vivo [10]. These mechanisms may provide a possible explanation for the increased macrophage migration observed in the presence of pancreatic cancer cells in the CGA.

A further strength of the CGA is the ability to simultaneously observe macrophage behavior and cancer cell dynamics within the same experimental well. This allowed quantification of macrophage recruitment in the presence or absence of tumor cells while also documenting lateral displacement of cancer cells over time. While this differs conceptually from the transwell assay, which primarily measures migration of a single cell population across a membrane, both approaches provide complementary information on cell motility under different experimental conditions. The use of distinct fluorescent labels enabled independent evaluation of both cell populations. Notably, the higher motility observed for PA-TU-8988T compared with PA-TU-8988S cells is consistent with previously reported differences in migratory behavior [32], supporting the robustness of the assay. In addition, the CGA format allows intra-well comparisons under identical culture conditions, which may be advantageous for future experimental designs.

Like any experimental system, the CGA has method-specific limitations. The assay is restricted to adherent cell types and may therefore not be suitable for studying non-adherent immune cell populations. Floating cells may occasionally enter the gap, potentially introducing variability. Manual removal of the ibidi© insert requires careful handling to avoid disruption of the gap. Moreover, quantification of migration within the first ~100 µm may be influenced by proliferation in highly proliferative cell lines such as RAW264.7. However, when performed under standardized conditions, these limitations do not substantially compromise the overall reliability of the assay.

It is also important to acknowledge that more advanced experimental platforms are available, including organ-on-a-chip systems [33], microfluidic devices [34], and 3D culture models [35,36], which offer higher physiological complexity. Organ-on-a-chip platforms replicate dynamic, in vivo-like microenvironments and enable detailed investigation of tumor–immune interactions. Microfluidic systems allow precise control of chemokine gradients and high-resolution analysis of chemotactic behavior. Three-dimensional culture models and tumor spheroids better recapitulate tumor architecture and heterogeneity compared with 2D systems. These advanced platforms provide complementary opportunities for investigating tumor–immune interactions. Within this methodological landscape, the CGA represents a reproducible, accessible, and versatile tool that adds value to the existing spectrum of in vitro migration assays [18,19,20,21]. Future adaptations of the CGA may enable its application to 3D culture models, providing a platform to study migration within matrix-rich, physiologically relevant microenvironments.

In conclusion, the Chamber Gap Assay represents a robust and reproducible method for studying tumor-induced macrophage migration in vitro. Its capacity to capture migration dynamics, provide spatial resolution, and enable morphological assessment supports its use as a complementary experimental approach in cancer immunology and translational research, particularly for studies addressing immune cell migration and macrophage phenotype modulation in the context of cancer.

## 5. Conclusions

The Chamber Gap Assay provides a practical and sensitive in vitro system for dissecting macrophage recruitment to pancreatic cancer cells. Its capacity to resolve migratory trajectories and associated morphological changes provides insight into how cancer cells influence macrophage behavior and may support the preclinical evaluation of TAM-targeting therapies. Future work incorporating chemokine receptor blockade and time-lapse imaging will be important to establish its utility for addressing defined mechanistic and therapeutic questions.

## Data Availability

All data supporting the findings of this study are included in the article and its Supplementary Materials. Additional raw imaging data and analysis files are available from the corresponding author upon request.

## Supplementary Materials

The following supporting information can be downloaded at: https://www.mdpi.com/article/10.3390/biology15030240/s1, Figure S1: Viability of peritoneal macrophages and THP-1–derived macrophages after 120 h in the Chamber Gap Assay. Table S1: Counts of THP-1 macrophages that migrated more than 500 µm, indicating ongoing migration efforts within the observational period.

## Appendix A

**Table A1:** Comparative characteristics of the Chamber Gap Assay (CGA) and the transwell assay for analyzing tumor-induced macrophage migration. Comparison of key features of the Chamber Gap Assay (CGA) and the transwell assay for studying tumor-induced macrophage migration. The table highlights differences in gradient formation, imaging capability, quantification, and technical considerations. The CGA provides spatially resolved, real-time analysis through fluorescence imaging, whereas the transwell assay offers a simple, high-throughput, endpoint-based approach. Each method delivers complementary insights and should be chosen according to the specific experimental objective.

Aspect	Chamber Gap Assay (CGA)	Transwell Assay
Cell type compatibility	Suitable for adherent tumor and immune cells	Applicable to both adherent and suspension cells
Gradient formation	Real-time gradient formation upon insert removal in shared microenvironment	Static gradient across membrane; limited control over gradient dynamics
Imaging and quantification	Spatial resolution via fluorescence microscopy and QuPath©; cell tracking across defined distances	Endpoint readout only; no spatial or directional information
Real-time visualization	Time-lapse imaging possible; allows dynamic analysis of migration kinetics and directionality	Not possible; only endpoint quantification
Migration depth resolution	Measures cell distribution across precise µm intervals in the gap	No spatial mapping; only migrated cell count below membrane
Proliferation control	Interpretation within first ~100 µm limited due to potential RAW264.7 proliferation near seeding front	Less affected by proliferation due to vertical design and short incubation time
Technical handling	Insert removal requires high precision to avoid disrupting cell boundaries	Simple setup with low technical variability
Artefact risk	Occasional floaters may remain in the gap despite washing and mimic migrated cells	Minimal; few artefacts when membrane integrity is preserved
Selective treatment	Enables pre-treatment of tumor or immune cells before barrier removal in the same dish	Independent treatment of upper and lower compartments possible
Quantification mode	Cell density per region, directionality	Total cell count or fluorescence intensity in lower well
Experimental flexibility	Supports dual labeling and live-cell imaging strategies	Compatible with label-based quantification; no imaging during migration
Cost and accessibility	Medium cost; requires ibidi inserts; fluorescence microscope needed (for real-time visualization -> with incubation mode for time lapse)	Medium cost; standard consumables and widely used
Throughput	Moderate; ideal for mechanistic and image-based studies	High; well suited for screening multiple experimental conditions
