# Supplementary material for: The Chamber Gap Assay Is a Simple and Sensitive In Vitro Method for Studying Pancreatic Cancer-Induced Macrophage Recruitment and Morphological Alteration
Source: Biology (Basel). 2026 Jan 28;15(3):240. doi: 10.3390/biology15030240 (PMC12897175; doi:10.3390/biology15030240)
Supplement: Supplementary file 1 [file biology-15-00240-s001.zip › Figure S1 Viability of peritoneal macrophages and THP-1–derived macrophages after 120 h in the Chamber Gap Assay..pdf]

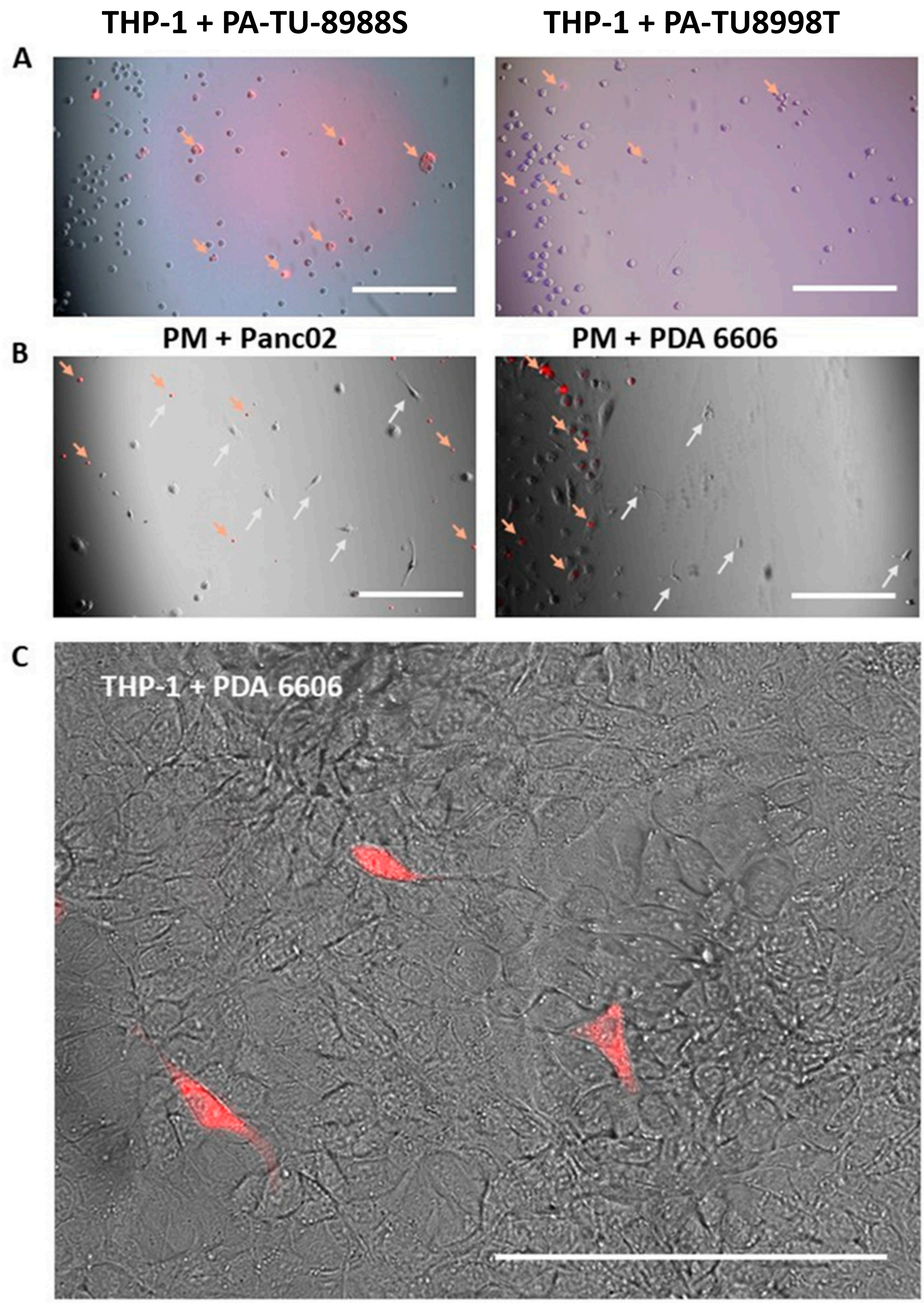


**Supplementary Figure.**
**Viability of peritoneal macrophages and THP-1–derived macrophages after 120 h in the Chamber Gap Assay.**
**(A)** Representative images of THP-1 macrophages co-cultured with PA-TU-8988S or PA-TU8998T cells at 120 h. Propidium iodide (PI) staining was used to identify non-viable cells (orange arrows). Occasional PI-positive cells are present, whereas migrating macrophages appear viable and adherent. **(B)** Representative images of peritoneal macrophages (PM) co-cultured with Panc02 or PDA6606 cells at 120 h. Few PI-positive cells are detectable (orange arrows), while the majority of cells remain viable. Typical macrophage morphology could be observed, with elongated PM in Panc02 cell co-culture and PM exhibiting multiple protrusions in PDA6606 cell co-culture. Scale bars = 100 µm. **(C)** High-magnification overlay of brightfield and CellTrace™ Red–labeled PM that migrated more than 500 µm toward the cancer cell compartment. Migrated macrophages appear sharply delineated, adherent, and viable, with no evidence of floating cells. Scale bar = 100 µm.
